# Metabolite profiling in retinoblastoma identifies novel clinicopathological subgroups

**DOI:** 10.1038/bjc.2015.318

**Published:** 2015-09-08

**Authors:** Sarah Kohe, Marie-Anne Brundler, Helen Jenkinson, Manoj Parulekar, Martin Wilson, Andrew C Peet, Carmel M McConville

**Affiliations:** 1 School of Cancer Sciences, University of Birmingham, Vincent Drive, Birmingham B15 2TT, UK; 2 Department of Histopathology, Birmingham Children's Hospital, Steelhouse Lane, Birmingham, B4 6NH, UK; 3 Department of Oncology, Birmingham Children's Hospital, Steelhouse Lane, Birmingham B4 6NH, UK; 4 Department of Ophthalmology, Birmingham Children's Hospital, Steelhouse Lane, Birmingham B4 6NH, UK

**Keywords:** retinoblastoma, magnetic resonance spectroscopy, taurine, choline, phosphocholine

## Abstract

**Background:**

Tumour classification, based on histopathology or molecular pathology, is of value to predict tumour behaviour and to select appropriate treatment. In retinoblastoma, pathology information is not available at diagnosis and only exists for enucleated tumours. Alternative methods of tumour classification, using noninvasive techniques such as magnetic resonance spectroscopy, are urgently required to guide treatment decisions at the time of diagnosis.

**Methods:**

High-resolution magic-angle spinning magnetic resonance spectroscopy (HR-MAS MRS) was undertaken on enucleated retinoblastomas. Principal component analysis and cluster analysis of the HR-MAS MRS data was used to identify tumour subgroups. Individual metabolite concentrations were determined and were correlated with histopathological risk factors for each group.

**Results:**

Multivariate analysis identified three metabolic subgroups of retinoblastoma, with the most discriminatory metabolites being taurine, hypotaurine, total-choline and creatine. Metabolite concentrations correlated with specific histopathological features: taurine was correlated with differentiation, total-choline and phosphocholine with retrolaminar optic nerve invasion, and total lipids with necrosis.

**Conclusions:**

We have demonstrated that a metabolite-based classification of retinoblastoma can be obtained using *ex vivo* magnetic resonance spectroscopy, and that the subgroups identified correlate with histopathological features. This result justifies future studies to validate the clinical relevance of these subgroups and highlights the potential of *in vivo* MRS as a noninvasive diagnostic tool for retinoblastoma patient stratification.

An estimated 5000–8000 new cases of retinoblastoma are diagnosed worldwide each year (40–50 in the United Kingdom), representing 4% of all childhood cancers (Abramson, 2005). Enucleation of the affected globe has long been considered the most effective treatment and is associated with survival rates approaching 100% in developed countries. Over recent years, there has been an increasing emphasis, however, on more conservative treatments to minimise loss of vision, problems associated with prosthesis fitting and maintenance, as well as the emotional and behavioural adjustments required in patients (van Dijk *et al*, 2007). This is particularly important for patients with heritable retinoblastoma (∼40% of the total) who have a high risk of bilateral disease.

Conservative treatments such as cryotherapy and laser therapy, in some cases combined with systemic chemotherapy, are now well established for retinoblastomas classified as low grade (grades A–C according to the International Intraocular Classification of Retinoblastoma) (Sastre *et al*, 2009). However, primary enucleation is more frequently the treatment of choice for high-grade tumours (grades D and E) as ∼25% of these will show adverse histopathological features following enucleation, for example, retrolaminar invasion of the optic nerve, massive choroidal invasion or involvement of the anterior chamber (Yousef *et al*, 2014). These features are considered to present a significant risk for extraocular metastasis (Shields and Shields, 2004) and thus patients also receive additional systemic chemotherapy post-enucleation. Delayed diagnosis and suboptimal therapy are associated with mortality rates of up to 70% in some developing countries as a consequence of tumour metastasis (Kivela, 2009).

More recently, the development of more effective methods for administration of chemotherapy using targeted intra-arterial or intra-vitreal approaches means that these are now being used increasingly as alternatives to enucleation. These methods have proved to be highly effective in the treatment of high-grade retinoblastoma, allowing globe salvage in 60–70% of cases (Munier *et al*, 2013; Shields *et al*, 2014). Nevertheless, a consequence of this development is that fewer globes undergo histopathological examination for high-risk features, which would otherwise prompt treatment with systemic chemotherapy to eliminate possible micrometastatic spread. Thus, it is possible that a cohort of children, who would have received chemotherapy for high-risk features if enucleated, will be significantly undertreated with intra-arterial/-vitreal chemotherapy and will face a greater risk of subsequent metastatic relapse. It has been reported, for example, that two patients in a series of 78 (2.5%) developed metastatic disease after intra-arterial chemotherapy (Gobin *et al*, 2011). At present, there are no reliable noninvasive methods to identify high-risk pathology before treatment (biopsy is contraindicated as it presents a risk of extraocular spread). There is an urgent need therefore to develop noninvasive tumour markers that could differentiate between retinoblastomas which could be treated conservatively, and those for which enucleation is still the safest option.

A potential solution to this problem is provided by the use of magnetic resonance spectroscopy (MRS), for the detection of tumour metabolites that may serve as biomarkers for tumour stratification. *Ex vivo* studies using high-resolution magic-angle spinning magnetic resonance spectroscopy (HR-MAS MRS) of resected tumour tissue have shown the utility of this method for tumour diagnosis and subtyping in brain, lung, pancreatic, gastric and colon cancers (Wilson *et al*, 2009a; Jung *et al*, 2014; Mirnezami *et al*, 2014; Rocha *et al*, 2014; Wang *et al*, 2014). Tumour metabolites have also been shown to correlate with tumour-specific genetic mutations, for example, IDH1 mutation in glioma, SDH and VHL mutations in paraganglioma and MYCN amplification in neuroblastoma (Peet *et al*, 2007; Imperiale *et al*, 2013; Esmaeili *et al*, 2014). Furthermore, and of particular relevance to retinoblastoma, is the demonstration that clinically important information may also be obtained noninvasively using *in vivo* MRS. This approach has been used to distinguish between different types of brain tumours and to predict survival of patients with these tumours (Orphanidou-Vlachou *et al*, 2013; Vicente *et al*, 2013; Wilson *et al*, 2013). *In vivo* MRS has also been used successfully to provide diagnostic and prognostic information about prostate, breast and pancreatic cancers (Yao *et al*, 2012; Selnaes *et al*, 2013; Vassiou *et al*, 2013).

It has been established that there is a high correlation between metabolites measured by HR-MAS MRS of *ex vivo* tissue and those measured using *in vivo* MRS (Wilson *et al*, 2009b). Therefore, in this study, we have assessed the utility of *ex vivo* HR-MAS MRS to identify metabolite biomarkers, which define tumour subtypes and which correlate with histopathology in retinoblastoma. This work provides the first demonstration that MRS may have significant value in clinical decision making in retinoblastoma and provides a foundation for further studies to develop *in vivo* MRS as a diagnostic tool for this tumour.

## Materials and methods

### Clinical samples

Frozen tumour tissue samples from eyes enucleated without prior treatment were obtained from 52 retinoblastoma patients (48 unilateral, 4 bilateral), treated at Birmingham Children's Hospital from 2004 to 2013. Tumours were selected based on the availability of frozen tissue. Consent was obtained for tissue banking for ethically approved research. This study was approved by the local Research Ethics Committee and the UK Children's Cancer and Leukaemia Group (CCLG) Biological Studies Committee.

### Histopathology analysis

Following removal of a portion of the tumour for HR-MAS MRS, the entire enucleated eye was processed into paraffin as part of standard clinical practice (Sastre *et al*, 2009), and every 5th–10th section stained with H&E for analysis. Analysis was carried out independently by two individuals, including a paediatric pathologist (MAB), across all the slides from each case (median: 17; range 13–22 slides per case). Criteria scored included the percentage of rosettes (Flexner–Wintersteiner (F–W) and/or fleurettes), the percentage of necrosis, level of apoptosis (localised patches were scored 1, and widespread scatter was scored 2), and the presence or absence of adverse pathology, including deep choroid invasion, retrolaminar optic nerve invasion and scleral invasion. As the presence of F–W rosettes and fleurettes is considered a marker of differentiation, the percentage of tumour occupied by rosettes/fleurettes was quantified on a graded scoring scale as a measure of differentiation. Tumours with <10% of the tumour occupied by rosettes were scored 1, those with 10–25% rosettes were scored 2, those with 26–50% rosettes were scored 3, whereas those with >50% rosettes were scored 4. Necrosis was defined by the areas of the tumour with cells that were shrunken and predominantly eosinophilic (stained pink) indicating DNA breakdown. Apoptotic cells were identified as cells with condensing and fragmented chromatin (stained blue), often accompanied by irregular cell size and shape and membrane degradation. Depth of invasion into the choroid was recorded as deep (>3 mm) or superficial (<3 mm), and optic nerve invasion was scored as prelaminar, intralaminar or retrolaminar based on the depth of intrusion into the optic nerve (Sastre *et al*, 2009). Histological analysis was undertaken on the whole paraffin-embedded tumour rather than on the tissue fragment used for HR-MAS MRS. However, a small subset of post-HR-MAS MRS tumours (*n*=10) were subsequently embedded in paraffin to confirm that the histological features of the HR-MAS MRS biopsy accurately reflected those of the whole tumour (Supplementary Table 3).

### High-resolution proton magnetic resonance spectroscopy

High-resolution magic-angle spinning magnetic resonance spectroscopy was undertaken at the Henry Wellcome Building for NMR Spectroscopy at the University of Birmingham. Frozen tumour tissue was trimmed to fit either a 12 or a 50 *μ*l zirconium rotor, weighed and 5 *μ*l of internal standard (3-trimethylsilylproponic-2,2,3,3-*d*4 acid sodium salt (TSP; Cambridge Biosciences, Cambridge, UK) was added before the rotor was topped up with deuterium water (Sigma-Aldrich, Dorset, UK). The average tissue weight of the samples was 21 ±7.8 mg (s.d.) and the minimum size suitable for obtaining good quality spectra was 5 mg. All samples were kept cold over dry ice during preparation to minimise tissue degradation. High-resolution magic-angle spinning magnetic resonance spectroscopy was carried out using a Bruker Avance spectrometer (Bruker Biospin, Coventry, UK) at a field strength of 500 MHz with a 4 mm three-channel HCD HR-MRS z-PFG band probe. Samples were spun at 4800 Hz at a temperature of 4 °C to minimise sample degradation. A pulse-acquire acquisition was used with 2 s of NOESY presaturation for water suppression and a repetition time of 4 s. Total acquisition time was either 17 or 34 min depending on sample size.

### Data analysis

High-resolution magic-angle spinning magnetic resonance spectroscopy data analysis of 52 retinoblastomas was performed using the Mnova NMR 8.2 software suite (2013; Mestrelab Research, Spain). Data were Fourier transformed, phased, baseline corrected and chemical shift referencing was performed relative to the creatine peaks at 3.03 p.p.m. The signal from spectral regions at 1.14 and 3.67 (representing ethanol contamination) was removed from all further analysis. Spectra were then binned into regions of 0.01 p.p.m., normalised to the total spectral area and the region between 2.6 and 4.0 p.p.m. subjected to multivariate analyses using principal component analysis (PCA). The region from 0 to 2.6 p.p.m. was not included in the multivariate analysis as the lipid and lactate signals dominate this region and vary greatly across the samples masking information from the small-molecule metabolites. This is an approach that has been used in several previous studies (Bathen *et al*, 2007). The scores from the principal components were then plotted. The metabolites that contribute to each PC score were assessed using the corresponding PC loadings. An unsupervised hierarchical clustering analysis using a Euclidian distance metric and average linkage was also undertaken using the same spectral data. All multivariate statistical methods were carried out using the Metaboanalyst 3.0 server (2012; The Metabolomics Innovation Centre, Edmonton, AB, Canada) (Xia *et al*, 2012).

Quantitation of relevant metabolites identified from loadings plots produced during multivariate analysis was undertaken in Mnova NMR for 50 retinoblastomas using the added TSMP as a concentration reference (Sitter *et al*, 2010; Moestue *et al*, 2011). Two cases from group 3 were excluded from quantification because of low signal from the TSMP reference peak. Metabolites were quantified by determining the peak area of the metabolite using a global spectral deconvolution (GSD) line-fitting algorithm and comparing to the peak area of the standard at known concentrations. This algorithm facilitates resolution of overlapping and hidden peaks in crowded areas of the spectrum (Bernstein *et al*, 2013). The proton number contributing to both the metabolite signal of interest and TSMP is also taken into account when performing quantification (Govindaraju *et al*, 2000). Peaks were assigned to particular metabolites manually using literature values and the Human Metabolome Database (Wishart *et al*, 2013). In spectral regions with overlapping peaks such as the lipid resonances, the signal from irrelevant peaks was subtracted from the GSD analysis before calculating the concentration. Concentration values were then adjusted according to sample weight and rotor size. Lipids were quantified as they are known to correlate with necrosis in tumours. Lipids were assigned according to Moestue *et al* (2011), and contributions from macromolecular resonances were excluded from lipid concentration values.

All statistical comparisons of metabolite concentrations and histological features between groups were undertaken in SPSS version 22 (IBM, Armonk, NY, USA) using the nonparametric Kruskall–Wallis test with significance set at *P*⩽0.05. This was followed by Mann–Whitney *U*-test pairwise comparisons as a *post hoc* test to identify group-specific differences. Correlations were examined using Spearman's rank correlation test or where appropriate, a point biserial correlation coefficient (rPB).

The Metabolite Pathway Analysis (MetPA) tool of the Metaboanalyst 3.0 server was used for network analysis of identified metabolites. MetPA uses pathway enrichment analysis together with the analysis of pathway topological characteristics to identify the most relevant metabolic pathways in the system under study. The pathway impact factor is calculated as the sum of the importance (centrality) measures of the metabolites of interest, normalised by the sum of the importance measures of all metabolites in the pathway (Xia and Wishart, 2010).

## Results

### HR-MAS MRS of retinoblastoma

**Figure 1:**
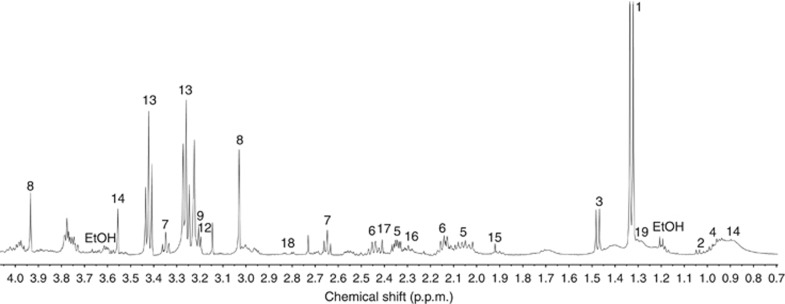
**HR-MRS spectrum typical of retinoblastoma.** Lactate (1), valine (2), alanine (3), leucine (4), glutamate (5), glutamine (6), hypotaurine (7), creatine (8), choline (9), glycerophosphocholine (10), phosphocholine (11), phosphoethanolamine (12), taurine (13), glycine (14), acetate (15), GABA (16), succinate (17), aspartate (18), lipid (19), ethanol contamination (*EtOH*).

High-resolution magic-angle spinning magnetic resonance spectroscopy of retinoblastoma has not been reported previously and therefore initial investigation focused on defining the range of metabolites detectable in these tumours. A total of 52 retinoblastomas (Supplementary Table 1) were analysed, revealing readily detectable levels of lactate, valine, alanine, leucine, glutamate, glutamine, hypotaurine, creatine, choline, phosphocholine, glycerophosphocholine, phosphoethanolamine, taurine, glycine, lipids, macromolecules, acetate, GABA, succinate and aspartate (Figure 1).

**Figure 2:**
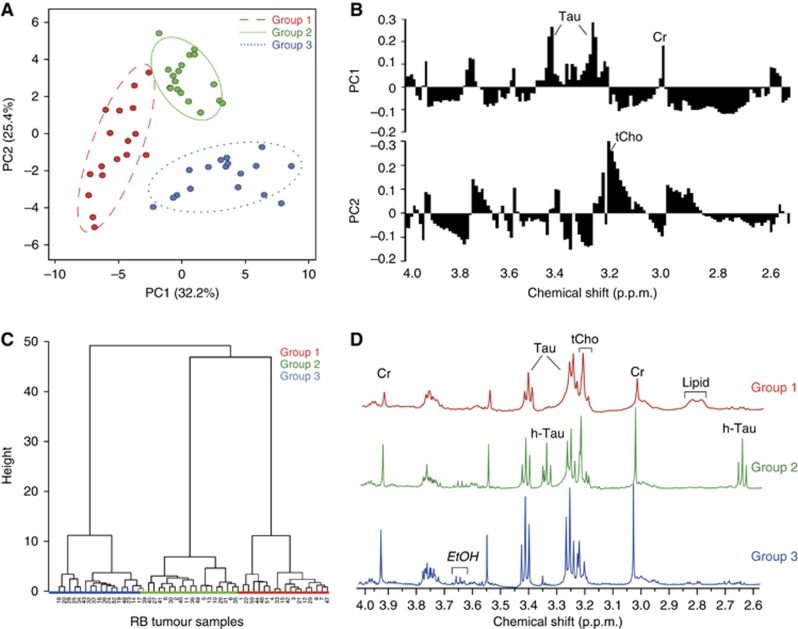
**Multivariate analysis of binned data from 2.6 to 4p.p.m.** (**A**) Unsupervised PCA showing three groups of retinoblastomas. Scores in PC1 and PC2 are bounded by 95% confidence intervals. (**B**) Principal component analysis loading plots showing the contribution of spectral bins in PC1 and PC2. Taurine-related bins are a major feature of PC1, and choline-related bins in PC2. (**C**) Unsupervised hierarchical clustering also identifies the same three groups of retinoblastomas. (**D**) Representative spectra from each group showing characteristic metabolite features. Group 1 has low taurine (Tau) and creatine (Cr), no hypotaurine (h-Tau) and a visible lipid presence at 2.8p.p.m. (lipid group 6), group 2 has high total-choline (tCho) relative to taurine, high Cr and high hypotaurine, whereas group 3 has high taurine and creatine relative to other metabolites and very little lipids.

Principal component analysis of the binned spectral region from 2.6 to 4 p.p.m. indicated that PC1 and PC2 together accounted for ∼58% of the variation across the tumours. Based on this analysis, separation of the tumours into three distinct groups was apparent (Figures 2A and B). An unsupervised hierarchical clustering analysis also showed the same classification of tumours into three separate groups (Figure 2C). Metabolites that map to the spectral bins contributing to this classification include taurine, creatine (PC1) and choline (PC2) (Figure 2B). Thus, group 1 retinoblastomas are characterised by low taurine and creatine with variable choline, group 2 by intermediate taurine and creatine but with high choline and group 3 by increased taurine and creatine, but lower choline (Figure 2D). Examination of PC3 identified hypotaurine as a further metabolite of interest, which provided good discrimination between group 1, where it is very low, and group 2, where it is particularly high (results not shown). Post-HR-MAS MRS histopathology review of a subset of 10 retinoblastomas confirmed that in all cases the tissue was composed entirely of tumour with no contamination from normal retina.

The primary analysis of spectral binned data maximises the information available for tumour classification but does not give precise information on metabolite levels. Therefore, concentrations of taurine, hypotaurine, choline, creatine and lipids were quantified and normalised as described in the Materials and Methods section. Calculation of mean values for each of the three retinoblastoma groups confirmed that in all cases concentrations of these metabolites showed significant differences between groups (detailed in Table 1 and summarised in Table 2).

**Figure 3:**
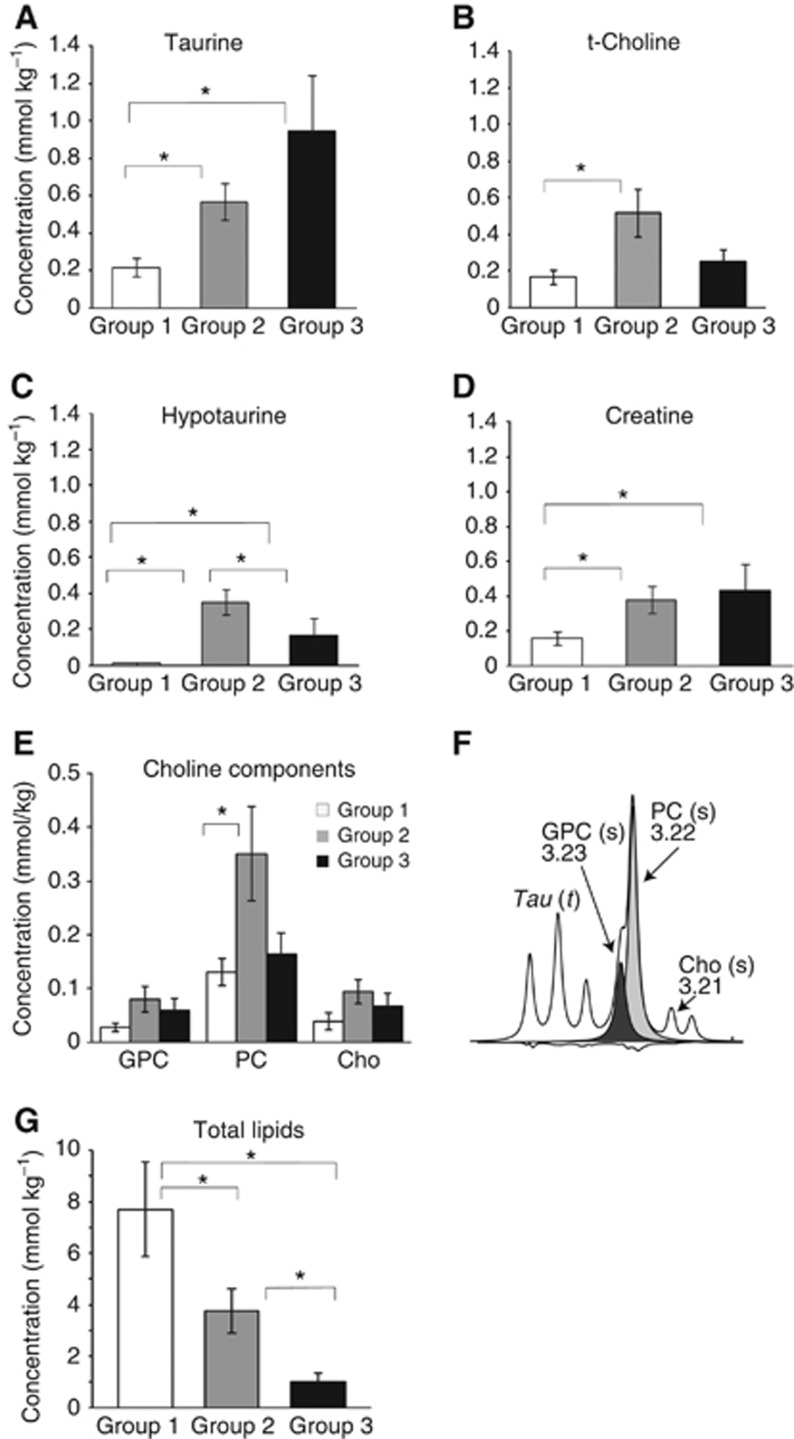
**Mean metabolite concentrations in retinoblastoma groups.** (**A**) Taurine, (**B**) total-choline, (**C**) hypotaurine, (**D**) creatine, (**E**) individual choline components and (**F**) choline containing region of the spectrum: GPC, glycerophosphocholine (s: singlet, black; t: triplet); PC, phosphocholine (s, grey); Cho, free choline. (**G**) Total lipids. *P⩽0.05; between groups statistical significance as determined by Mann–Whitney *U*-test *post hoc* comparisons.

The most notable differences were observed for taurine metabolites. Both taurine and hypotaurine were significantly lower in group 1 compared with groups 2 and 3 (Figure 3). Amounts were variable in the latter two groups, with significantly higher hypotaurine in group 2 (2.2-fold increase relative to group 3) and a trend towards higher taurine in group 3 (1.7-fold increase). Creatine concentrations showed a similar pattern to taurine (2.4- and 2.7-fold increases in groups 2 and 3 relative to group 1). An inverse relationship between lipids and taurine was apparent, with group 1 retinoblastomas having lipid concentrations 2.1- and 7.7-fold higher compared with groups 2 and 3, respectively. Group 2 retinoblastomas were characterised by very high total-choline concentrations. Measurements of individual choline metabolites, that is, choline, phosphocholine and glycerophosphocholine, indicated that phosphocholine was largely responsible for the 3.1-fold increase in total-choline in group 2 relative to group 1.

Pathway analysis of the quantified metabolites identified the taurine/hypotaurine metabolic pathway as showing the most significant difference between the three retinoblastoma subgroups (impact factor=0.41; FDR-adjusted *P*-value=0.005). Metabolite set enrichment analysis of the pathway identified high hypotaurine and high taurine concentrations as significant features of groups 2 and 3, respectively. The glycerophospholipid pathway was also significantly different between groups (impact factor=0.08; FDR-adjusted *P*-value=0.002), with phosphocholine and glycerophosphocholine contributing most to this difference (Supplementary Figure 1). This pathway analysis further emphasises the very distinctive metabolic profiles for each of the retinoblastoma subgroups and suggests that metabolic pathways involving taurine and choline metabolites are important in retinoblastoma biology.

### Histopathology of retinoblastoma and its relationship to the metabolite-based tumour classification

**Figure 4:**
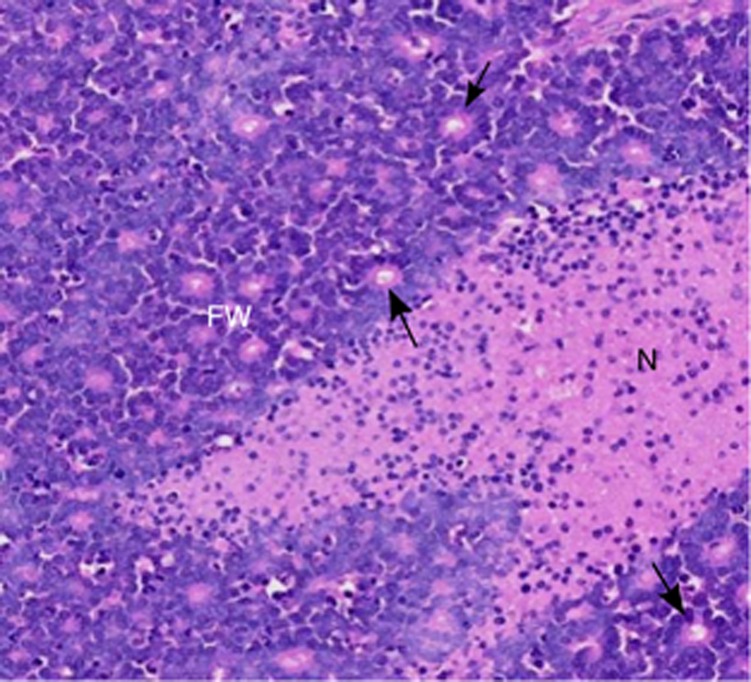
**H&E histology of a representative retinoblastoma.** FW, Flexnor–Wintersteiner rosettes characteristic of differentiated retinoblastoma (the arrows point to individual examples of well-defined rosettes). N, necrotic tumour (stained pink).

To investigate the clinicopathological significance of variations in the retinoblastoma metabolome, all retinoblastomas were also assessed for histopathological parameters. These included measures of tumour viability (necrosis and apoptosis), differentiation (F–W rosettes; Figure 4) and invasiveness (invasion of the choroid or optic nerve). Each parameter was assessed across multiple tissue sections from each retinoblastoma to maximise the accuracy of the analysis. Independent analysis of the tissue samples used for HR-MAS MRS was also undertaken for a subset of cases and confirmed that the HR-MAS MRS tissue was representative of the tumour as a whole (Supplementary Table 3).

**Figure 5:**
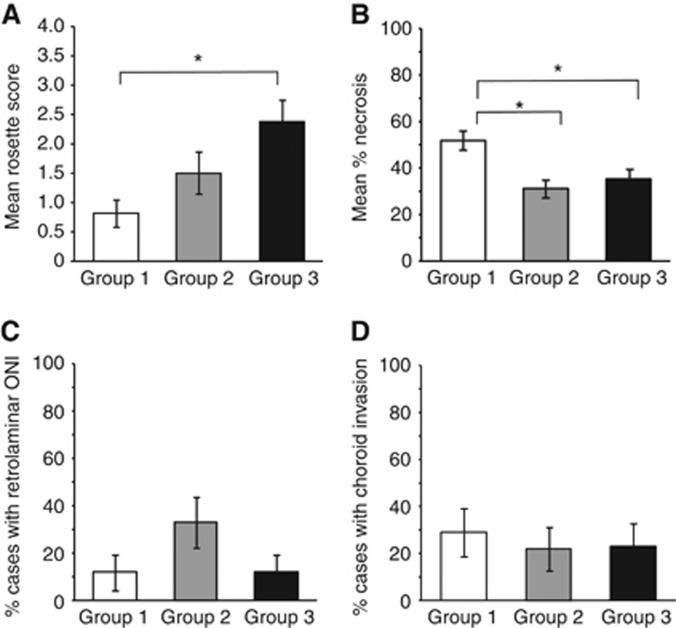
**Histopathology of retinoblastoma metabolic groups.** (**A**) Differentiation, (**B**) necrosis, (**C**) retrolaminar optic nerve invasion and (**D**) deep choroid invasion.

One of the most significant observations from this analysis was the variation in the level of differentiation among the different retinoblastoma groups (*P*=0.015). Most group 1 tumours were relatively undifferentiated (13 out of 17, 76% with <10% of the tumour containing F–W rosettes), whereas many group 3 tumours showed much higher levels of differentiation (35% of tumours with >50% rosettes) (Figure 5 and Supplementary Table 2). The level of differentiation showed a significant positive correlation with taurine concentration (*P*=0.002). Necrosis (but not apoptosis) also varied significantly between groups (*P*=0.002) and was higher in the undifferentiated group 1 tumours (52% necrotic cells) compared with either group 2 (31%, *P*=0.0005) or group 3 (35%, *P*=0.01). The level of necrosis was positively correlated with total lipids (*P*=0.0005) but negatively correlated with taurine (*P*=0.009), creatine (*P*=0.005), hypotaurine (*P*=0.03), total-choline (*P*=0.02) and phosphocholine (*P*=0.05) (Supplementary Table 2).

Retrolaminar optic nerve invasion was more frequent in group 2 retinoblastomas (6 out of 18, 33%) compared with that in the other groups (2 out of 17, 12% in both groups 1 and 3). Although this distribution did not reach statistical significance, it is of interest that retrolaminar optic nerve invasion was significantly correlated with higher total-choline (*P*=0.004) and phosphocholine (*P*=0.001) concentrations, which were also a characteristic feature of group 2 retinoblastomas. No differences were observed in the frequency of deep choroid invasion between groups.

## Discussion

Retinoblastoma differs from most other tumour types in that diagnosis, staging and treatment decisions must rely on clinical observations and imaging, without the benefit of input from histopathology or molecular pathology. This presents a major problem for the delivery of personalised treatment, particularly as this is complicated by the need not only to ensure patient survival but also to attempt globe salvage and preservation of vision. In this study, we show for the first time that magnetic resonance spectroscopy potentially is a valuable diagnostic tool in retinoblastoma. High-resolution magic-angle spinning magnetic resonance spectroscopy provides a detailed readout of the tumour metabolome, variations of which are expected to reflect the upstream genetic mutations, which drive tumour development and progression. Our results demonstrate that HR-MAS MRS can be used to discriminate between retinoblastoma subgroups, and in addition that clinically relevant aspects of tumour biology such as differentiation and optic nerve invasion are correlated with metabolic parameters.

The metabolites that contributed most to discrimination between retinoblastoma groups included taurine, hypotaurine, phosphocholine, creatine and lipids. Although no single metabolite was exclusively present in any retinoblastoma group, metabolite concentrations showed consistent differences between groups, and when considered in combination, provided group-specific profiles. Group 1 retinoblastomas, for example, were defined by very low taurine, little or no hypotaurine and low creatine, as well as significantly elevated lipids. Group 2 retinoblastomas had the highest levels of phosphocholine and also hypotaurine. Group 3 retinoblastomas were characterised by high taurine and very low lipid concentrations.

The observation that taurine was highest in group 3 retinoblastomas and that this group was also the most differentiated, as determined by the presence of F–W rosettes and fleurettes (markers of retinal and photoreceptor differentiation; Eagle, 2013) is consistent with reports that taurine has an important role in retinal and particularly photoreceptor development and function. Several studies have demonstrated that taurine depletion is associated with photoreceptor degeneration (Froger *et al*, 2014). Conversely, taurine contributes to the *in vitro* generation of putative rod and cone photoreceptors from embryonic stem cells (Osakada *et al*, 2008). These effects may be mediated at least in part by neuromodulatory effects of taurine. Taurine is structurally similar to the neurotransmitters glycine and GABA and has been shown to activate glycine receptors to stimulate rod photoreceptor development (Ripps and Shen, 2012). Photoreceptor differentiation is also influenced by taurine-upregulated gene 1, a non-coding RNA that modulates the expression of photoreceptor-specific genes in the retina (Young *et al*, 2005).

Other cellular functions of taurine include osmoregulation, calcium modulation and suppression of inflammation, and it also has antioxidant properties. In addition, it is important in maintaining the normal respiratory chain function (Schaffer *et al*, 2014), a factor that is critical in retinal photoreceptor cells, as this is one of the most energetically demanding cell types in the body (Wong-Riley, 2010; Reidel *et al*, 2011). Consistent with this, we have shown previously that more differentiated retinoblastomas express many genes required for photoreceptor function and also show significant upregulation of pathways associated with mitochondrial function (Kapatai *et al*, 2013). The mechanisms regulating taurine levels in retinoblastoma are unclear but may involve either regulation of its synthesis from the precursor hypotaurine and/or regulation of uptake from the extracellular environment, mediated by the taurine transporter SLC6A6.

Taurine has been implicated as a tumour marker in a number of different cancer types. The fact that it is both increased (e.g. colorectal, breast, prostate cancers) and decreased (oesophageal carcinomas) relative to normal tissue (Swanson *et al*, 2003; Yang *et al*, 2013; Mirnezami *et al*, 2014) is perhaps not surprising in view of its multiple functions. However, it is of interest that in breast cancer, higher taurine levels are associated with improved survival (Cao *et al*, 2012; Choi *et al*, 2012), paralleling the observation of increase taurine levels in retinoblastomas with a lower frequency of retrolaminar optic nerve invasion. In the CNS taurine promotes differentiation along a neuronal, rather than a glial pathway (Ripps and Shen, 2012), and this is consistent with the detection of increased levels of taurine in medulloblastomas but not in astrocytomas or ependymomas (Moreno-Torres *et al*, 2004; Davies *et al*, 2008). Increased taurine levels have also been reported in neuroblastoma, a tumour of the peripheral nervous system (Wilson *et al*, 2009a).

These observations suggest that taurine metabolism in retinoblastoma is closely linked to aspects of normal retinal/neural function. In contrast, alteration of total-choline/phosphocholine metabolism may be regarded as a hallmark of malignant transformation and is a much more general characteristic of many different tumour types (Righi *et al*, 2009; Glunde *et al*, 2011; Ward *et al*, 2013). Phosphocholine is both a precursor and a breakdown product of cellular membranes and its metabolism is important for cellular proliferation. Total-choline and phosphocholine are also increased in response to hypoxia (Glunde *et al*, 2011). There is evidence to suggest that choline metabolism is regulated by oncogenic signalling, involving, for example, RAS and PI3K/AKT, as well as transcription factors including JUN, MYC, MYCN and HIF1, which regulate enzymes and transporters in the choline pathway (Ronen *et al*, 2001; Peet *et al*, 2007; Glunde *et al*, 2011). Increased total-choline levels have been associated with aggressiveness in breast, prostate and brain tumours (Peet *et al*, 2007; Righi *et al*, 2009; Glunde *et al*, 2011). This is consistent with our findings in retinoblastoma that increased total-choline and phosphocholine were significantly correlated with retrolaminar optic nerve invasion (*P*⩽0.004). The total-choline : creatine ratio has also been used as a measure of malignancy and combines measures of proliferative capacity (total-choline) with energy metabolism (tumours with high metabolic activity tend to have depleted energy stores and low creatine) (Martinez-Bisbal and Celda, 2009). Using this ratio, retinoblastomas may be classified in increasing order of malignancy: group 3 (total-choline : creatine ratio: 0.6)<group 1 (1.1)<group 2 (1.4).

Group 1 retinoblastomas showed the highest levels of lipids. Only mobile lipids (not membrane-associated lipids) are visible in MR spectra, and consequently these are likely to originate from cytoplasmic lipid droplets. The presence of lipid droplets has been correlated with necrosis and/or apoptosis (Abramson, 2005), and this is consistent with the observation of increased necrosis in this retinoblastoma group. However, these retinoblastomas frequently also contained abundant mitoses (results not shown), and increased necrosis may be a response to hypoxic conditions, as might be found where there is extensive tumour growth within the vitreous. Therefore, these retinoblastomas may have reduced capacity to maintain growth when the nutrient supply is limiting.

Our results suggest that metabolomic investigation of retinoblastoma has the potential to provide clinically useful information about tumour characteristics. It is important, however, to consider the challenges involved in replicating this work using noninvasive *in vivo* MRS in patients. *In vivo* MRS has lower resolution compared with HR-MAS MRS; nevertheless, the key metabolites of interest that we have identified *ex vivo* have already been quantified in *in vivo* MRS studies. In paediatric brain tumours, for example, significant changes in mean metabolite concentrations in the range of 1.3- to 5-fold for taurine, lipid, creatine and choline accurately discriminate between tumour types (Davies *et al*, 2008). It is likely, therefore, that differences of this magnitude in taurine, choline and lipids could also be detected *in vivo* in retinoblastoma. Previous work from our group has shown that successful and accurate *in vivo* quantification can be performed with software such as LC model and Tarquin (Reynolds *et al*, 2006; Davies *et al*, 2008) and that these values correlate well with *ex vivo* metabolite concentrations from the same samples (Wilson *et al*, 2009b).

Continuing technical developments in the MRI field will also contribute to improved MRS. The use of small surface coils, for example, is of great value where there is a requirement for high spatial resolution in a small field of view. These have already been used succesfully to investigate extrascleral extension in uveal melanoma (Tartaglione *et al*, 2014) and have also been shown to be of value for the detection of postlaminar optic nerve invasion and choroidal invasion in children with retinoblastoma (Sirin *et al*, 2015). The development of scanners that operate at higher magnetic field strengths will also increase the sensitivity of MRI/MRS. Currently, clinical scanners operate at 1.5 or 3.0 Tesla, but studies on enculeated eyes using ultrahigh-field (7 Tesla) magnetic resonance microscopy have demonstrated the potential for the differential diagnosis of intraocular masses (Falke *et al*, 2013), and the feasibility of 7 T MRI *in vivo* has also been demonstrated (Richdale *et al*, 2009; Graessl *et al*, 2014).

In conclusion, the results of this study confirm previous genetic investigations showing heterogeneity among retinoblastomas, with loss of differentiation associated with a more invasive phenotype (Kapatai *et al*, 2013). Our metabolomic studies provide additional information about the biological differences between these retinoblastoma groups and suggest that even within less differentiated tumours, different metabolite profiles may allow further stratification, for example, retinoblastomas with increased lipids may be more necrotic and have less potential for progressive growth compared with retinoblastomas that have lower lipids, high phosphocholine and a high total-choline : creatine ratio.

The potential to stratify retinoblastomas noninvasively, using magnetic resonance spectroscopy, is of immense clinical significance in view of the increasing use of intra-arterial chemotherapy. Intra-arterial chemotherapy can produce marked tumour shrinkage, and the possibility for preservation of vision is also a very major consideration for patients (Shields *et al*, 2014). However, IAC does not treat systemic micrometastatic disease and thus very careful selection of patients is required to minimise the risk of potentially fatal extraocular metastasis. The findings reported here will require further validation to ascertain the full clinical relevance of these retinoblastoma subgroups; nevertheless, the results of this study indicate that metabolomic investigation using magnetic resonance spectroscopy could make a significant contribution to risk assessment in retinoblastoma patients, if as shown successfully in other studies, *in vivo* results correlate well with *ex vivo* data(Martinez-Bisbal and Celda, 2009; Wilson *et al*, 2009b). Future research must focus therefore on further validation of our findings and replicating our *ex vivo* data using *in vivo* magnetic resonance spectroscopy in a clinical setting, as well as determining the underlying genetic and biological basis of the metabolomic subgroups.

## Figures and Tables

**Table 1:** Metabolite concentrations in RB groups

**Rb group**	**Metabolites**					
	**Taurine**	**Hypotaurine**	**t-Choline**	**Phosphocholine**	**Creatine**	**Total lipid**
Group 1	0.22±0.04	0.01±0.006	0.17±0.05	0.13±0.04	0.16±0.04	7.70±1.84
Group 2	0.57±0.11	0.35±0.10	0.52±0.13	0.35±0.09	0.38±0.08	3.74±0.85
Group 3	0.95±0.30	0.16±0.07	0.29±0.07	0.16±0.07	0.43±0.14	1.00±0.35
Kruskall–Wallis *P*-value^a^	0.005	0.0001	0.008	0.007	0.016	0.0003
*Post hoc* significance^b^	G1 *vs* G3 *P*=0.002	G1 *vs* G2 *P*=0.0001	G1 *vs* G2 *P*=0.003	G1 *vs* G2 *P*=0.003	G1 *vs* G2 *P*=0.006	G1 *vs* G2 *P*=0.015
	G1 *vs* G2 *P*=0.006	G1 *vs* G3 *P*=0.013			G1 *vs* G3 *P*=0.048	G1 *vs* G3 *P*=0.0003
		G2vG3 *P*=0.03				G2 *vs* G3 *P*=0.002

Abbreviations: ANOVA=analysis of variance; Rb=retinoblastoma.

a Nonparametric ANOVA for significance of difference between groups.

b Values ⩽0.05 are considered significant.

**Table 2:** Relative differences in metabolite concentrations between retinoblastoma groups

**Metabolite**	**Group 1**	**Group 2**	**Group 3**
Taurine	Low	Intermed	High
Hypotaurine	Very low	High	Intermed
Creatine	Low	High	High
t-Choline	Low	High	Low
t-Lipid	High	Intermed	Low
