# Supplementary material for: Metabolite profiling in retinoblastoma identifies novel clinicopathological subgroups
Source: Br J Cancer. 2015 Sep 8;113(8):1216–24. doi: 10.1038/bjc.2015.318 (PMC4647873; doi:10.1038/bjc.2015.318)
Supplement: Supplementary Figure S1 [file bjc2015318x1.pdf]

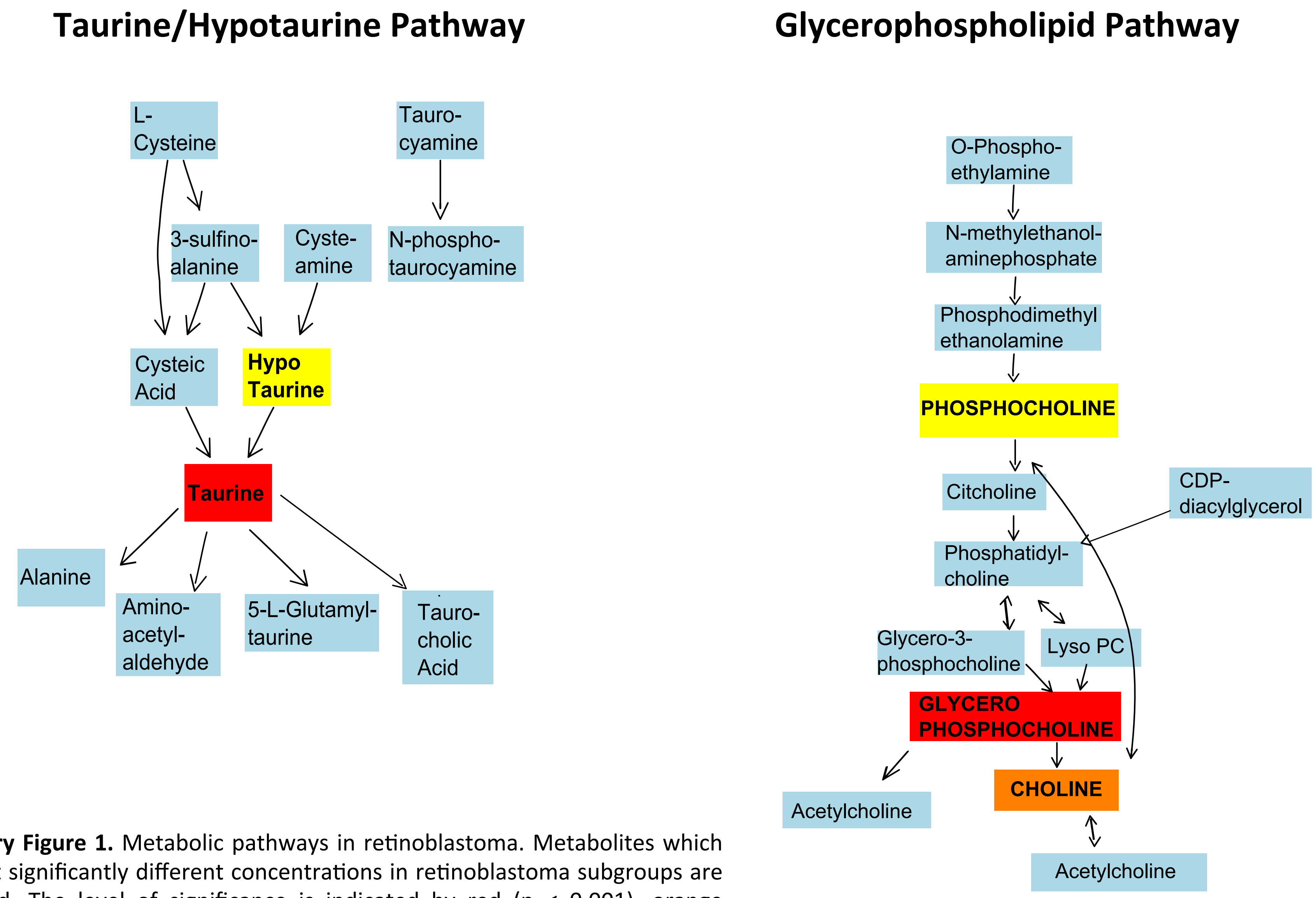

Supplementary Figure 1. Metabolic pathways in retinoblastoma. Metabolites which are present at significantly different concentrations in retinoblastoma subgroups are shown in bold. The level of significance is indicated by red ($p < 0.001$), orange ($p<0.02$) or yellow ($p < 0.05$).
